# Regulation of cyclic AMP response-element binding-protein (CREB) by G_q/11_-protein-coupled receptors in human SH-SY5Y neuroblastoma cells

**DOI:** 10.1016/j.bcp.2007.10.015

**Published:** 2008-02-15

**Authors:** Elizabeth M. Rosethorne, Stefan R. Nahorski, R.A. John Challiss

**Affiliations:** Department of Cell Physiology and Pharmacology, University of Leicester, Henry Wellcome Building, Lancaster Road, Leicester LE1 9HN, UK

**Keywords:** CREB, cyclic AMP response-element binding-protein, ERK, extracellular signal-regulated kinase, GPCR, G-protein-coupled receptor, IBMX, 3-isobutyl-1-methylxanthine, mACh, muscarinic acetylcholine, MAPK, mitogen-activated protein kinase, PDBu, phorbol 12,13-dibutyrate, PKA, cyclic AMP-dependent protein kinase, PKC, protein kinase C, PLC, phospholipase C, Cyclic AMP response-element binding-protein (CREB), Muscarinic acetylcholine receptor, Bradykinin receptor, SH-SY5Y cells, Intracellular calcium concentration [Ca^2+^]_i_, Calmodulin, Ca^2+^/calmodulin-dependent protein kinase, Protein kinase C, c-Fos

## Abstract

Human SH-SY5Y neuroblastoma cells have been used to investigate mechanisms involved in CREB phosphorylation after activation of two endogenously expressed G_q/11_-protein-coupled receptors, the M_3_ muscarinic acetylcholine (mACh) and B_2_ bradykinin receptors. Stimulation with either methacholine or bradykinin resulted in maximal increases in CREB phosphorylation within 1 min, with either a rapid subsequent decrease (bradykinin) to basal levels, or a sustained response (methacholine). Inhibitor studies were performed to assess the involvement of a number of potential kinases in signalling to CREB phosphorylation. Removal of extracellular Ca^2+^, inhibition of Ca^2+^/calmodulin-dependent protein kinase II and down-regulation of protein kinase C (PKC) resulted in reduced CREB phosphorylation after both M_3_ mACh and B_2_ bradykinin receptor activation. In contrast, inhibition of MEK1/2 by U0126 resulted in significantly reduced CREB phosphorylation levels after B_2_ bradykinin, but not M_3_ mACh receptor activation. In addition, we demonstrate that maintained phosphorylation of CREB is necessary for CRE-dependent gene transcription as the M_3_ mACh, but not the B_2_ bradykinin receptor activates both a recombinant CRE-dependent reporter gene, and the endogenous c-Fos gene. These data highlight the involvement of multiple, overlapping signalling pathways linking these endogenous G_q/11_-coupled metabotropic receptors to CREB and emphasize the importance of the duration of signalling pathway activation in converting a CREB phosphorylation event into a significant change in transcriptional activity.

## 1 Introduction

Adaptive responses in neurons often involve changes in gene expression, which allow long-term phenotypic changes such as differentiation of precursor cells, or synaptic strengthening of mature neurones. Such changes in gene expression occur in the CNS via the activity of neuronal transcription factors such as the cyclic AMP response-element binding-protein (CREB), nuclear factor of activated T cells (NF-AT) and nuclear factor-κ enhancer binding-protein (NF-κB). CREB activity is induced in a vast array of cell types [1], in neuronal cells these signals may be in the form of synaptic transmissions, neurotransmitters or neurotrophic factors. CREB is activated by the phosphorylation of a specific serine residue (Ser-133) within its kinase-inducible domain (KID), in response to a variety of extracellular signals [2]. This leads to the activation of so-called CREB kinases, which include the cAMP-dependent protein kinase (PKA; [3]), Ca^2+^/calmodulin-dependent protein kinases [4–6] ribosomal S6 kinases [7], extracellular signal-regulated kinases (ERK; [8,9]), protein kinase B [10] and protein kinase D [11]. In addition, protein kinase C (PKC) activation has been linked to CREB phosphorylation in numerous studies, but only occasionally has a direct phosphorylation been demonstrated [12]. Thus, it is likely that CREB phosphorylation by PKC often involves one or more intermediate kinases.

Signalling events leading to phosphorylation of CREB following synaptic activity have been well characterized [13–17]. Less well understood however, are the specific mechanisms leading to CRE-dependent gene transcription following G-protein-coupled receptor (GPCR) activation. Another layer of complexity is added if the variety of gene products expressed as a result of activation of a single transcription factor such as CREB is taken into account. To allow this specificity of response, strict regulatory events are required. One of the ways in which this specificity is accomplished is through differing Ca^2+^ signals. Firstly, the spatial characteristics of increases in intracellular Ca^2+^ can differentially activate either cytoplasmic or nuclear effector molecules. Hardingham et al. [18] demonstrated that increases in nuclear Ca^2+^ concentration result in the activation of CREB and hence CRE-dependent gene expression. A second signalling pathway that resulted in increases in cytoplasmic Ca^2+^ concentration led to the activation of serum-response-element (SRE)-dependent gene expression. In addition, the site of Ca^2+^ entry may be important, such that Ca^2+^ ions entering at dendritic loci, as opposed to the cell body, may cause a different complement of proteins to be activated.

The type of Ca^2+^ signal created can also influence the response achieved. The release of Ca^2+^ from intracellular stores can occur as a peak or peak-and-plateau type response, or an oscillatory response. Ca^2+^ oscillations have been shown to increase the efficiency of gene expression by lowering the effective Ca^2+^ threshold for transcription factor activation [19]. The temporal profile of the Ca^2+^ signal determines the magnitude of response obtained, so that although short bursts of Ca^2+^ elevation are sufficient to initiate gene transcription, prolonged signalling is required for a maximum response [20].

To investigate the roles of some of these differences in Ca^2+^ signalling, we have utilized the neuronal SH-SY5Y cell-line that shares many characteristics with foetal sympathetic ganglion cells [21]. This cell-line expresses at least two endogenous G_q/11_-coupled GPCRs, the M_3_ muscarinic acetylcholine (mACh), and B_2_ bradykinin receptors [22]. Activation of these two receptors results in downstream signalling cascades, and activation of kinases capable of phosphorylating CREB. For this reason, the SH-SY5Y cell-line represents a suitable cell model to investigate mechanisms involved in GPCR coupling to CREB within a neuronal context.

## 2 Materials and methods

### 2.1 Materials

All chemicals and reagents were purchased from Sigma–Aldrich (Poole, UK) unless otherwise indicated. Antibodies raised to ^133^Ser-phosphorylated CREB and total CREB were purchased from New England BioLabs (Beverly, USA); antibody raised against active phospho-ERK (pERK1/2), Glo-lysis buffer and Steady-Glo reagent were purchased from Promega (Madsion, USA); Fura-2-AM was purchased from Molecular Probes (Cambridge, UK); glass coverslips were purchased from BDH-Merck (Poole, Dorset); thapsigargin, phorbol 12,13-dibutyrate, Ro 31-8220, U0126 and antibody raised against c-Fos were purchased from Calbiochem (Nottingham, UK); western blotting equipment and Tris/glycine/sodium dodecyl sulfate running buffer were from BioRad (Hertfordshire, UK); nitrocellulose transfer membrane was from Schliecher & Schuell (London, UK); acrylamide was from Flowgen (Leicestershire, UK); enhanced chemiluminescence and hyperfilm were purchased from Amersham Pharmacia (Little Chalfont, UK); all tissue culture reagents were purchased from Invitrogen (Paisley, UK).

### 2.2 Cell culture

Human SH-SY5Y neuroblastomas were maintained in minimum essential medium with Earl's salts (without l-glutamate) supplemented with foetal bovine serum (10%), penicillin (100 IU mL^−1^), streptomycin (100 μg mL^−1^) and l-glutamine (2 mM), at 37 °C, 5% CO_2_. For experiments, cells were harvested using trypsin/EDTA and seeded on to multiwell plates in medium composed as above. All experimental manipulations with viable cells were performed at 37 °C.

### 2.3 Preparation of cell lysates

Cell lysates were prepared as described previously [23,24]. Prior to agonist stimulation, monolayers were washed twice and incubated for 30 min at 37 °C in freshly oxygenated Krebs-Henseleit buffer (KHB (in mM); 10 HEPES, 118 NaCl, 4.7 KCl, 1.2 MgSO_4_, 1.2 KH_2_PO_4_, 4.2 NaHCO_3_, 1.3 CaCl_2_, 11.7 glucose, pH 7.4). Drug additions were made directly to the KHB at concentrations and time-points indicated in the figure legends, and after indicated treatments plates were placed on ice. Cell stimulations were terminated by rapid removal of the agonist-containing buffer, followed by two washes with ice-cold phosphate buffered saline (PBS (in mM); 140 NaCl, 2.68 KCl, 8.1 Na_2_HPO_4_, 1.47 KH_2_PO_4_, pH 7.4), and solubilization using relevant lysis procedures (see below).

For pCREB and c-Fos determination cells were lysed by addition of 150 μL 1× SDS sample buffer (62.5 mM Tris/HCl, 2% SDS, 10% glycerol, 0.1% bromophenol blue, 50 mM dithiothreitol (DTT), pH 7.4), immediately scraped off the plate and transferred to a microfuge tube. Cell lysates were sonicated for 20 s to shear DNA and reduce sample viscosity, boiled for 5 min and centrifuged at 20,000 × *g* for 1 min at 4 °C.

For pERK determination, cells were solubilized in lysis buffer (20 mM Tris/HCl, 1% Triton-X100, 10% glycerol, 137 mM NaCl, 2 mM EDTA, 25 mM β-glycerophosphate, 1 mM Na_3_VO_4_, 1 mM phenylmethylsulfonyl fluoride, 10 mg ml^−1^ leupeptin, 10 mg ml^−1^ aprotinin, pH 7.4) and centrifuged (33,000 × *g*, 10 min, 4 °C) to remove insoluble material. Aliquots of supernatant were removed and added to equivalent volumes of 2× sample buffer containing 50 mM DTT, boiled for 5 min and centrifuged (20 000 *g*, 1 min, 4 °C).

### 2.4 Western blotting analysis

Samples for western blotting (25 μL) were separated by 10% SDS-polyacrylamide gel electrophoresis using the BioRad minigel system. Proteins were electro-transferred to nitrocellulose membrane using the BioRad semi-dry blotter apparatus according to manufacturer's instructions. Following electro-transfer, the membranes were blocked for non-specific binding at room temperature (20 °C) for 1 h in Tris-buffered saline (50 mM Tris/HCl, 150 mM NaCl, pH 7.6) containing 0.1% Tween-20 (TBS-T), supplemented with 5% non-fat milk. After blocking, membranes were washed in TBS-T, and incubated overnight at 4 °C with the either polyclonal pCREB-specific antibody (1:1000 dilution in 5% milk/TBS-T), the polyclonal anti-active pERK1/2 (1:5000 in 1% BSA/TBS-T), or the polyclonal anti-c-Fos antibody (1 μg mL^−1^ in 5% milk/TBS-T). Primary antibodies were detected for 1 h at room temperature with a secondary antibody (goat anti-rabbit; 1:1000 dilution in 5% milk/TBS-T) conjugated to horseradish peroxidase. This was followed by chemiluminescence detection using ECL-plus reagent and exposure to HyperFilm™ (Amersham Life Science). To ensure equal protein loading across the gel, membranes were submerged in stripping buffer (62.5 mM Tris/HCl, 100 mM 2-mercaptoethanol, 2% (w/v) SDS, pH 6.7), and incubated at 50 °C for 40 min, and re-probed with a polyclonal control CREB antibody, or the anti-tubulin antibody, followed by detection with the same secondary antibody.

### 2.5 Measurement of Ins(1,4,5)P_3_ mass

Cells were grown to confluency in 24-well multiwell plates for the measurement of Ins(1,4,5)P_3_ mass, which was performed as described previously [25]. In brief, cells were washed with KHB and incubated at 37 °C for 30 min. Agonist additions were made directly to the KHB. Reactions were stopped by the addition of an equal volume (250 μL) of ice-cold 1 M trichloroacetic acid (TCA), and following neutralization the Ins(1,4,5)P_3_ content was determined.

### 2.6 Single-cell imaging of intracellular Ca^2+^

Changes in the levels of [Ca^2+^]_i_ were followed in Fura-2AM (2 μM, 1 h) loaded cells using a Nikon Diaphot inverted epifluorecence microscope. Cells were seeded on 25 mm glass coverslips, and incubated overnight at 37 °C. Prior to imaging, cells were washed with Krebs-Henseleit buffer and incubated with 2 μM Fura-2 AM for 1 h, in the dark at room temperature. After washing, coverslips were maintained in KHB and mounted on the microscope stage. Drugs were made up in KHB and perfused over cells for the required time intervals. Cells were excited at 340 and 380 nm using a Spectramaster II monochromator (Perkin-Elmer Life Sciences) and sequential images were captured via a charge-coupled device camera (Photonic Science) using Merlin2000 data acquisition system (Perkin-Elmer Life Sciences). Changes in cytosolic Ca^2+^ were measured by converting the 340/380 nm fluorescence ratio (after background subtraction) to [Ca^2+^]_i_ using the method of Grynkiewicz et al. [26].

### 2.7 Recombinant pCRE-LUC reporter gene assay

After overnight incubation on 24-well multiwell plates, cells were co-transfected with 0.5 μg mL^−1^ pCRE-luc reporter using Lipofectamine. Cells were incubated with Lipofectamine and DNA (3:1 ratio) for 5 h in growth medium devoid of all supplements, after which time medium was changed to complete growth medium. On the third day of growth cells were washed, and incubated overnight in serum-free medium. On the day of the experiment, cells were treated with agonist in serum-free medium for times indicated in figure legends, followed by further incubation in serum-free medium to make 6 h incubation in total. Cells were harvested using 150 μL Glo-lysis buffer for 5 min, after a brief wash with Dulbecco's PBS. Fifty microlitres of cell lysates and 50 μL of Steady-Glo reagent were added to wells of a black, 96-well plate, and incubated at room temperature for 5 min, after which time luminescence was quantified.

### 2.8 Quantification and data analysis

Immunoreactivities were quantified using the Syngene GeneGnome System with Gene Tools software. Data were fitted as sigmoidal concentration-response curves and statistical differences between datasets determined by one-way analysis of variance (ANOVA) for multiple comparisons, followed by Bonferroni's multiple-range test at *p* < 0.05, using GraphPad Prism 4 software (GraphPad, San Diego, CA).

## 3 Results

### 3.1 GPCR-mediated CREB phosphorylation in SH-SY5Y cells

**Fig. 1:**
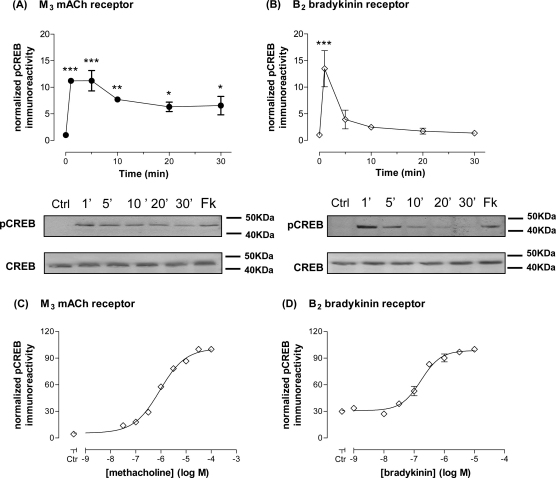
Time-courses and concentration-dependencies for receptor-mediated phosphorylation of the transcription factor CREB in SH-SY5Y cells. Time-dependent increases in phospho-CREB immunoreactivity after stimulation of cells with either methacholine (1 μM; A) or bradykinin (300 nM; B). A representative immunoblot for phospho-CREB and total CREB is shown beneath each graph (Ctr, buffer addition; 1′–30′, times (in min) of exposure to agonist; Fk, response to addition of 10 μM forskolin/500 μM IBMX for 10 min). MCh activation resulted in significant (^*^p < 0.05; ^**^p < 0.01; ^***^p < 0.001) increases in phospho-CREB between 1 and 30 min stimulation, while activation by bradykinin resulted in a significant increase in phospho-CREB only after 1 min of stimulation. Concentration-dependent increases in phospho-CREB immunoreactivity were assessed at a 5 min time-point for M_3_ mACh receptor (C) or B_2_ bradykinin receptor (D) stimulation with the indicated concentrations of agonist. For each individual experiment, data have been normalized to the increase in phospho-CREB observed in the presence of a maximal concentration of agonist (100 μM methacholine and 30 μM bradykinin, respectively). For each panel, data are shown as means ± S.E.M. for at least 3 independent experiments.

The ability of M_3_ mACh and B_2_ bradykinin receptors to cause the phosphorylation of CREB was initially assessed by challenging SH-SY5Y cells with methacholine (MCh; 1 μM) or bradykinin (300 nM) for 0–30 min. Activation of either receptor did not affect total CREB protein expression, however an early, robust (approximately 10–15-fold) increase in CREB phosphorylation was observed in response to either MCh or bradykinin. The temporal profile for CREB phosphorylation differed for the two receptors being studied: MCh caused a rapid increase in CREB phosphorylation, which was maximal within 1 min (approximately 12-fold-over-basal) and declined to a steady-state plateau (approximately 6-fold-over-basal) after 5 min (Fig. 1A). In contrast, while bradykinin causes a similar initial increase in CREB phosphorylation (approximately 14-fold-over-basal at 1 min), a subsequent rapid decrease was observed such that 5–30 min values were not significantly greater than basal levels (Fig. 1B). Assessment of concentration-dependencies for agonist-stimulated CREB phosphorylation revealed EC_50_ values of 0.89 μM (pEC_50_, 6.05 ± 0.06) and 168 nM (pEC_50_, 6.76 ± 0.08) for MCh and bradykinin, respectively (Fig. 1C and D). With respect to the CREB phosphorylation response to MCh stimulation, prior addition of atropine (5 μM) prevented this response; similarly, addition of atropine 5 min after MCh also caused a rapid return to baseline within 5 min of antagonist addition (data not shown).

### 3.2 Analysis of second messenger signalling in SH-SY5Y cells

**Fig. 2:**
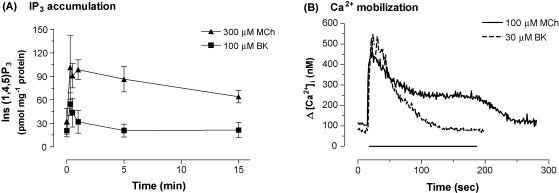
Time-courses for receptor-mediated increases in Ins(1,4,5)P_3_ and Ca^2+^ mobilization. Time-dependent increases in Ins(1,4,5)P_3_ (A) or [Ca^2+^]_i_ (B) after stimulation of cells with either MCh (300 μM) or bradykinin (30 μM). In (A), data are shown as means ± S.E.M. for at least 3 independent experiments; in (B) representative traces are shown.

Addition of the MCh (300 μM) resulted in an approximately 3-fold increase in levels of Ins(1,4,5)P_3_. Similar to increases in phospho-CREB, this response demonstrated peak-plateau type characteristics that were sustained for at least 15 min (Fig. 2A). Both the peak-and-plateau phases of the response were shown to be concentration-dependent (data not shown), with EC_50_ values of 30 μM (pEC_50_, 4.52 ± 0.30) and 7.7 μM (pEC_50_, 5.12 ± 0.23) for peak (15 s) and plateau (5 min) phases, respectively. Stimulation of cells with bradykinin (10 μM) resulted in a transient, 2-fold increase in Ins(1,4,5)P_3_ (Fig. 2A). Again, this response was concentration-dependent, with an EC_50_ of 103 nM (pEC_50_, 6.98 ± 1.21) (data not shown).

In addition to the classical Ins(1,4,5)P_3_ pathway, the M_3_ mACh and B_2_ bradykinin receptors are also capable of activating cyclic AMP accumulation. In the case of the M_3_ mACh receptor this occurs via a pertussis toxin-insensitive mechanism [27], suggesting the activation of Ca^2+^-dependent adenylate cyclases. Here, activation of the two receptors using MCh (300 μM, 5 min) or bradykinin (10 μM, 5 min) only resulted in a significant increase in cyclic AMP levels when cells were pre-treated with the phosphodiesterase inhibitor 3-isobutyl-1-methylxanthine (IBMX; 500 μM) (data not shown).

Previous work [22,28] has demonstrated that $\text{C}{\text{a}}_{\text{i}}^{\text{2+}}$ signalling through the M_3_ mACh and B_2_ bradykinin receptors have different temporal profiles. In our hands, addition of MCh (100 μM, 3 min) resulted in a prolonged Ca^2+^ signal that was sustained throughout the duration of receptor activation (Fig. 2B). When agonist was washed from cells, the [Ca^2+^]_i_ returned to basal levels. Challenge of SH-SY5Y cells with bradykinin (10 μM, 3 min) also resulted in a peak [Ca^2+^]_i_, although basal levels were regained after approximately 2 min, indicating a lack of a sustained [Ca^2+^]_i_ elevation (Fig. 2B). MCh elicited an increase in the $\text{C}{\text{a}}_{\text{i}}^{\text{2+}}$ signal with an EC_50_ of 0.8 μM (pEC_50_, 6.11 ± 0.12), bradykinin elicited a peak increase in $\text{C}{\text{a}}_{\text{i}}^{\text{2+}}$ signal with an EC_50_ of 13 nM (pEC_50_, 7.90 ± 0.39) (data not shown).

### 3.3 Effect of membrane depolarization on CREB phosphorylation in SH-SY5Y cells

**Fig. 3:**
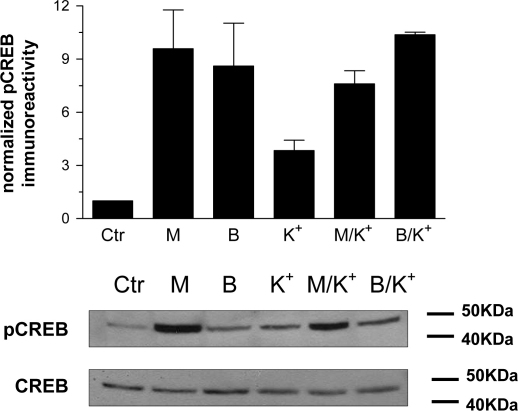
Effects of membrane depolarization on agonist-induced increases in CREB phosphorylation in SH-SY5Y cells. The effect of increasing extracellular [K^+^] (from 4.7 to 44.7 mM) *per se*, or in combination with MCh (1 μM) or bradykinin (300 nM) addition on phospho-CREB immunoreactivity was assessed in confluent SH-SY5Y cell monolayers. Representative immunoblots for phospho-CREB and total CREB are shown beneath the histogram (Ctr, buffer only addition; M, MCh (1 μM); B, bradykinin (300 nM); K^+^, +40 mM K^+^; M/K^+^ and B/K^+^, K^+^-depolarization in presence of respective agonist). Histogram data are shown as means ± S.E.M. for at least 3 independent experiments.

The effect of K^+^-depolarization on CREB phosphorylation *per se*, and on receptor-mediated CREB phosphorylation was assessed in SH-SY5Y cells. Depolarization-induced Ca^2+^ entry by addition of 40 mM K^+^ resulted in an approximately 4-fold increase in pCREB (Fig. 3). However, the peak M_3_ mACh or B_2_ bradykinin receptor-mediated CREB phosphorylation responses were not significantly affected by concurrent depolarization (Fig. 3).

### 3.4 Effect of altering Ca^2+^ gradients on receptor-mediated CREB phosphorylation

**Fig. 4:**
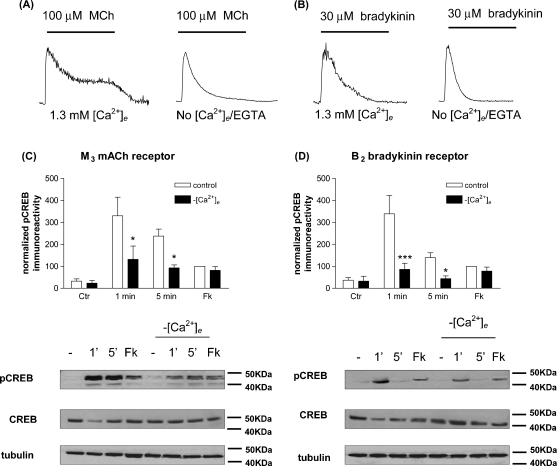
Extracellular Ca^2+^-dependency of agonist-stimulated CREB phosphorylation in SH-SY5Y cells. Representative [Ca^2+^]_i_ traces for responses stimulated by MCh (100 μM; A) and bradykinin (30 μM; B) in Fura-2AM-loaded cells. Experiments were performed in normal KHB (1.3 mM $\text{C}{\text{a}}_{\text{e}}^{\text{2+}}$) or under conditions where $\text{C}{\text{a}}_{\text{e}}^{\text{2+}}$ was depleted prior to agonist addition. The effects of the manipulation of Ca^2+^ gradients on agonist-stimulated changes in phospho-CREB immunoreactivity were assessed for MCh (1 μM; 1 or 5 min; C) or bradykinin (300 nM; 1 and 5 min; D). For each panel representative phospho-CREB, total CREB and γ-tubulin blots are shown. Responses were normalized with respect to the response to forskolin (10 μM)/IBMX (500 μM) addition for 10 min (Fk) in normal KHB (=100%) and are shown as means ± S.E.M. for at least 3 separate experiments. Statistically significant differences between agonist-stimulated responses under the different Ca^2+^ conditions are indicated as ^*^p < 0.05 or ^***^p < 0.001.

It has been demonstrated previously that CREB is a Ca^2+^-dependent transcription factor in many cell systems [2,29]. For this reason, the effect of removing extracellular Ca^2+^ ($\text{C}{\text{a}}_{\text{e}}^{\text{2+}}$) on receptor-mediated CREB phosphorylation was assessed (Fig. 4). Removal of $\text{C}{\text{a}}_{\text{e}}^{\text{2+}}$ from the perfusion medium and addition of EGTA (100 μM) resulted in removal of the sustained phase of the MCh-stimulated [Ca^2+^]_i_ response (Fig. 4A) and a marked inhibition of receptor-mediated CREB phosphorylation (by 61 ± 13% (at 1 min) and 76 ± 13% (at 5 min) compared to responses in the presence of normal (1.3 mM) $\text{C}{\text{a}}_{\text{e}}^{\text{2+}}$; Fig. 4C)). Combined $\text{C}{\text{a}}_{\text{e}}^{\text{2+}}$ removal/EGTA addition plus thapsigargin treatment (to empty $\text{C}{\text{a}}_{\text{i}}^{\text{2+}}$ stores) resulted in a complete abolition of the MCh-stimulated [Ca^2+^]_i_ response, but no greater inhibition of the receptor-mediated CREB phosphorylation response (data not shown).

Although removal of $\text{C}{\text{a}}_{\text{e}}^{\text{2+}}$ only slightly altered the bradykinin-stimulated [Ca^2+^]_i_ response (Fig. 4B) this manipulation had a marked inhibitory effect on CREB phosphorylation stimulated by the B_2_ bradykinin receptor (62 ± 13% (at 1 min) and 74 ± 10% (at 5 min) inhibitions compared to responses elicited in normal KHB; Fig. 4D)). Again, combined $\text{C}{\text{a}}_{\text{e}}^{\text{2+}}$ removal/EGTA addition plus thapsigargin treatment resulted in complete abolition of the bradykinin-stimulated [Ca^2+^]_i_ response, but no greater inhibition of the receptor-mediated CREB phosphorylation response (data not shown). Removal of $\text{C}{\text{a}}_{\text{i}}^{\text{2+}}$ (and/or $\text{C}{\text{a}}_{\text{e}}^{\text{2+}}$) did not significantly affect forskolin/IBMX-stimulated CREB phosphorylation (Fig. 4C and D).

### 3.5 Role of Ca^2+^/calmodulin-dependent protein kinases

**Fig. 5:**
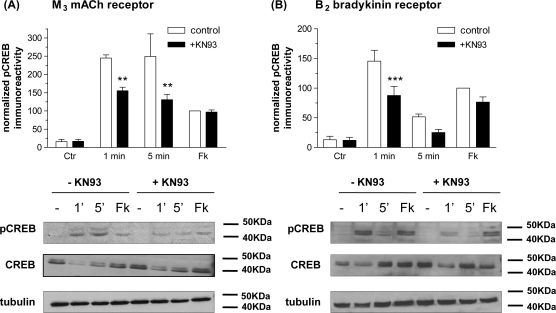
Effects Ca^2+^/CaM-kinase inhibition by KN93 on agonist-stimulated CREB phosphorylation in SH-SY5Y cells. KN93 (10 μM) was added to confluent SH-SY5Y cell monolayers 30 min prior to MCh (1 μM, 1 and 5 min; A) or bradykinin (300 nM, 1 and 5 min; B). Cells were also stimulated with forskolin (10 μM)/IBMX (500 μM) for 10 min (Fk). Representative immunoblots for phospho-CREB, total CREB and γ-tubulin are shown beneath each histogram. Data are normalized to the forskolin/IBMX response (=100%) in the absence of the inhibitor and are shown as means ± S.E.M. for at least 3 independent experiments. Statistically significant differences caused by KN93 are indicated as ^*^p < 0.05 and ^***^p < 0.001.

To investigate further the involvement of Ca^2+^ and the Ca^2+^-dependent CaM-kinases in CREB phosphorylation, the effect of pre-treatment of cells with the CaM-kinase II inhibitor KN93 was assessed with respect to M_3_ mACh and B_2_ bradykinin receptor-stimulated CREB phosphorylation (Fig. 5). In the presence of KN93 (10 μM, 30 min pre-incubation) both M_3_ mACh and B_2_ bradykinin receptor-stimulated increases in CREB phosphorylation were attenuated (40 ± 5 and 51 ± 22% reductions after MCh (Fig. 5A); 50 ± 14 and 79 ± 22% reductions after bradykinin (Fig. 5B), at 1 and 5 min, respectively). Forskolin/IBMX-stimulated CREB phosphorylation was unaffected in cells treated with KN93 (Fig. 5A and B). Similar results were obtained using KN62 an alternative CaM-kinase II inhibitor (data not shown).

### 3.6 Role of protein kinase C

**Fig. 6:**
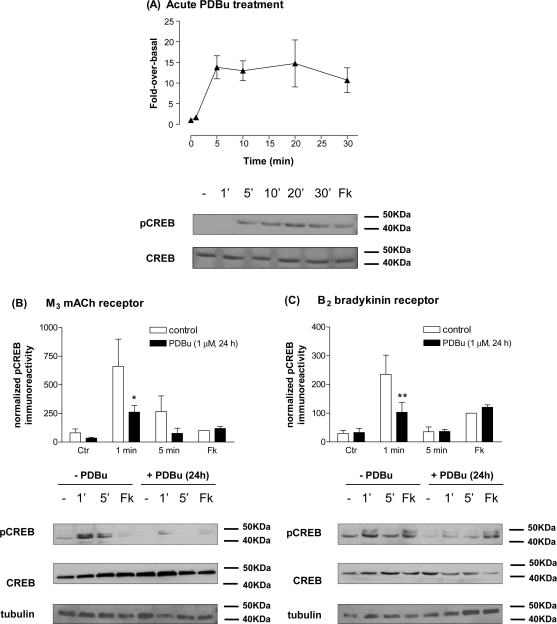
Dependency of agonist-stimulated CREB phosphorylation on PKC activity in SH-SY5Y cells. The phorbol ester, PDBu (1 μM) causes a time-dependent increase in phospho-CREB immunoreactivity (panel A). Effects of chronic phorbol ester treatment (1 μM PDBu; 24 h treatment) on CREB phosphorylation stimulated by MCh (1 μM; 1 and 5 min; B) or bradykinin (300 nM; 1 and 5 min; C). Cells were also stimulated with forskolin (10 μM)/IBMX (500 μM) for 10 min (Fk). Representative immunoblots for phospho-CREB, total CREB and γ-tubulin are shown beneath each histogram. Data, normalized to the forskolin/IBMX response (=100%) in the absence of PDBu treatment, are shown as means ± S.E.M. for at least 3 independent experiments. Statistically significant changes caused by chronic PDBu treatment are indicated as ^**^p < 0.01.

In addition to the CaM-kinases, increases in [Ca^2+^]_i_ and diacylglycerol (DAG) will activate a variety of protein kinase C (PKC) isoenzymes. SH-SY5Y cells have been shown to express abundantly the conventional PKC isoenzymes α and βI, and direct activation by the phorbol ester PDBu results in PKC translocation to the plasma membrane [22,30]. Here, we have demonstrated that addition of PDBu (1 μM) to SH-SY5Y cells to stimulate directly conventional (and novel) PKC isoenzymes results in a marked (approximately 14-fold at 5 min) and sustained increase in phospho-CREB levels (Fig. 6A). To assess the dependency of MCh- and bradykinin-stimulated CREB phosphorylation on PKC activation, we chronically treated SH-SY5Y cells with PDBu (1 μM, 20–24 h) to cause a down-regulation of classic and novel isoenzymes. The PKC down-regulation protocol resulted in significant reductions in M_3_ mACh and B_2_ bradykinin-stimulated phospho-CREB (MCh (1 μM), 58 ± 12%; bradykinin (300 nM), 57 ± 10% reductions compared to control conditions at 1 min; Fig. 6B and C). It should be noted that, in contrast to a previous report [31], we observed no change in the morphology of the SH-SY5Y cells, and M_3_ mACh receptor expression and coupling to phosphoinositide turnover was unchanged following PDBu treatment (data not shown). Nevertheless, as chronic phorbol ester treatment has been reported to initiate differentiation of these cells [32], we also used acute PKC inhibition with Ro 31-8220 (10 μM, 30 min pre-incubation) to confirm that the effects we observed using PDBu were attributable to PKC inhibition. This protocol resulted in qualitatively similar inhibitory effects on agonist-stimulated CREB phosphorylation to those caused by PKC down-regulation (data not shown).

### 3.7 Involvement of cyclic AMP-dependent protein kinase (PKA) and phosphoinositide 3-kinase (PI3-kinase)-dependent pathways

**Fig. 7:**
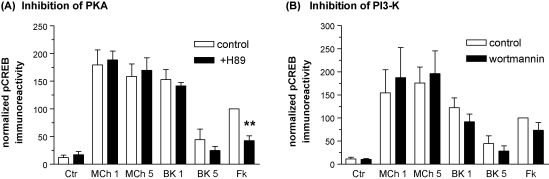
Dependency of agonist-stimulated CREB phosphorylation on PKA or PI3-kinase activities in SH-SY5Y cells. Effects of the PKA inhibitor H89 (10 μM; 30 min pre-treatment) or the PI3-kinase inhibitor wortmannin (100 nM; 30 min pre-treatment) on CREB phosphorylation stimulated by MCh (1 μM; 1 and 5 min) or bradykinin (300 nM; 1 and 5 min). Cells were also stimulated with forskolin (10 μM)/IBMX (500 μM) for 10 min (Fk). Representative immunoblots for phospho-CREB and total CREB are shown beneath each histogram. Data, normalized to the forskolin/IBMX response (=100%) in the absence of respective inhibitor treatment, are shown as means ± S.E.M. for at least 3 independent experiments. Statistically significant changes in the forskolin/IBMX response caused by H89 treatment are indicated as ^*^p < 0.05, ^**^p < 0.01.

Stimulation of M_3_ mACh and B_2_ bradykinin receptors in SH-SY5Y cells causes concentration-dependent, modest (2–3-fold) increases in cyclic AMP levels which are either sustained (pEC_50_ MCh (at 5 min), 6.54 ± 0.09) or transient (pEC_50_ bradykinin (at 1 min), 8.12 ± 0.13). However, these responses required the presence of the phosphodiesterase inhibitor IBMX (500 μM) and in its absence no significant cyclic AMP accumulation could be measured for either agonist. Nevertheless, to assess the involvement of a cyclic AMP-dependent pathway in M_3_ mACh or B_2_ bradykinin receptor linkage to CREB, PKA activity was inhibited using H89 (10 μM, 30 min pre-treatment). H89 had no effect on either MCh- or bradykinin-stimulated CREB phosphorylation (Fig. 7A), but this inhibitor did significantly attenuate responses stimulated by forskolin (in the presence of IBMX).

It has been demonstrated previously that receptor activation can lead to the phosphorylation of PKB/Akt in SH-SY5Y cells, and that PKB phosphorylation can be inhibited using the PI3-kinase inhibitor, wortmannin [33]. Pre-treatment of cells with wortmannin (100 nM, 30 min) to inhibit the PI3-kinase/PKB/Akt pathway did not significantly affect CREB phosphorylation stimulated by either M_3_ mACh or B_2_ bradykinin receptor activation (Fig. 7B).

### 3.8 Involvement of the extracellular signal-regulated kinase (ERK) pathway

**Fig. 8:**
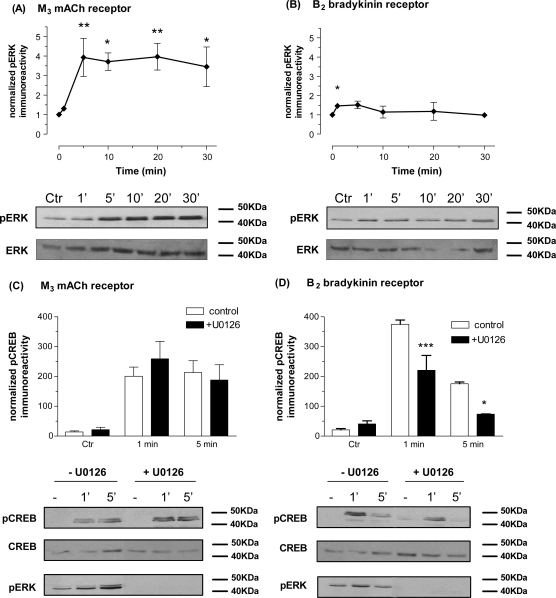
Dependency of agonist-stimulated CREB phosphorylation on ERK activity in SH-SY5Y cells. Time-courses of ERK1/2 phosphorylation stimulated by MCh (1 μM; A) or bradykinin (300 nM; B). Representative immunoblots for phospho-ERK1/2 and total ERK1/2 are shown beneath each histogram. Statistically significant increases in ERK1/2 phosphorylation are indicated as ^*^p < 0.05, ^**^p < 0.01. The effects of the MEK1/2 inhibitor U0126 (10 μM, 30 min pre-treatment) on MCh (1 μM; at 1 and 5 min; C) or bradykinin (300 nM; at 1 and 5 min; D) stimulated increases in phospho-CREB immunoreactivity. Representative immunoblots for phospho-CREB, total CREB and phospho-ERK1/2 are shown beneath each histogram. Data, normalized to the forskolin/IBMX response (=100%) in the absence of inhibitor, are shown as means ± S.E.M. for at least 3 independent experiments. Statistically significant effects of U0126 on CREB phosphorylation are indicated as ^*^p < 0.05, ^***^p < 0.001.

Consistent with previous reports [34,35] activation of M_3_ mACh receptors caused an approximately 4-fold increase in phospho-ERK immunoreactivity in SH-SY5Y cells, which are sustained for at least 30 min (Fig. 8A). In contrast, activation of the B_2_ bradykinin receptor resulted in only a transient and more modest (1.5-fold) increase in phospho-ERK (Fig. 8B). As has been observed previously in the SK-N-SH cell-line [36], inhibition of ERK1/2 activation using the MEK inhibitor U0126 (10 μM, 30 min pre-treatment) had no effect on M_3_ mACh receptor-stimulated CREB phosphorylation (Fig. 8C). However, in the presence of U0126 the bradykinin-stimulated increase in phospho-CREB was significantly attenuated (by 51 ± 5 and 81 ± 5% at 1 and 5 min, respectively) (Fig. 8D).

### 3.9 Additivity of effects of extracellular Ca^2+^ omission, PKC and ERK pathway inhibition on receptor-driven CREB phosphorylation

The effects of combining (i) extracellular Ca^2+^ depletion (KHB -Ca^2+^/+EGTA, see Section 2) and PKC inhibition (by chronic phorbol ester-induced down-regulation), and (ii) PKC inhibition and ERK pathway inhibition (using the MEK1/2 inhibitor U0126) have also been assessed. Combining extracellular Ca^2+^ depletion with PKC inhibition markedly inhibited the abilities of MCh (91 ± 5% inhibited at 1 min) and bradykinin (89 ± 9% inhibited at 1 min) to stimulate CREB phosphorylation to a greater extent than either treatment alone (data not shown; see Figs. 4 and 6). In contrast, combining PKC and ERK pathway inhibition did not result in any greater inhibition than that seen for PKC inhibition alone (for bradykinin at 1 min: PKC inhibition, 57 ± 10%; for U0126, 51 ± 5; combined PKC/ERK inhibition, 41 ± 15%). This non-additivity would be expected for the MCh-stimulated phospho-CREB response where no role for ERK can be discerned (see Fig. 8), however, with respect to the response to bradykinin, these data suggest that the ERK/PKC effects may be redundant or lie on a common pathway leading to CREB phosphorylation.

### 3.10 Do M_3_ mACh and B_2_ bradykinin receptor-mediated effects lead to transcriptional activation?

**Fig. 9:**
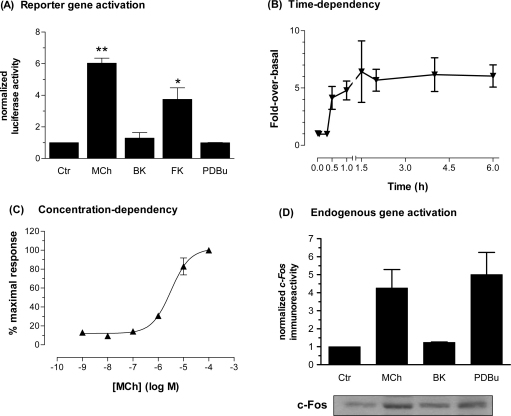
Agonist-stimulated effects on pCRE-luciferase reporter gene activity and c-Fos in SH-SY5Y cells. Sub-confluent SH-SY5Y cells were transfected with the pCRE-luciferase reporter construct as described in *Materials and methods*. Cells were treated with MCh (100 μM), bradykinin (30 μM), PDBu (1 μM) or forskolin (10 μM)/IBMX (500 μM) for 1 h, followed by further 5 h incubation in serum-free medium (A). Time-dependency of increases in CREB-dependent luciferase activity after stimulation of cells with MCh (100 μM) for the times indicated (B). Concentration-dependency of CREB-dependent luciferase activity after 1 h stimulation with MCh at concentrations indicated (C). In addition, cells were stimulated with MCh (100 μM), bradykinin (30 μM) or PDBu (1 μM) for 2 h prior to lysis and c-Fos determination (D; see Section 2). A representative immunoblot showing c-Fos immunoreactivity is shown below the histogram. Data are normalized to the luciferase activity or c-Fos immunoreactivity detected after assay buffer addition alone, and are presented as means ± S.E.M. for 3 independent experiments each performed in duplicate.

In the experiments up to this point, Ser-133 phosphorylation of CREB has been used as a convenient index of the activation of CRE-mediated gene transcription. To more directly investigate this we have utilized a recombinant reporter construct encoding the pCRE-luciferase gene. SH-SY5Y cells were transiently transfected with the pCRE-Luc reporter and after 72 h were stimulated with either MCh (100 μM) or bradykinin (30 μM). Stimulation of cells with MCh resulted in a 6-fold increase in luciferase activity, while bradykinin did not cause a significant increase relative to vehicle-stimulated cells (Fig. 9A). Similar to the CREB phosphorylation assay used in previous results, forskolin treatment resulted in a significant increase (4-fold) in luciferase activity only when cells were pre-treated with IBMX (500 μM). Furthermore, phorbol ester treatment failed to significantly increase CREB-driven luciferase activity. For MCh, the increase in reporter activity was shown to be both time- (Fig. 9B) and concentration-dependent (pEC_50_, 5.84 ± 0.63; Fig. 9C).

In addition to the recombinant reporter gene, we also assessed the activation of the endogenous CRE-dependent c-Fos gene. Cells were stimulated with either MCh (100 μM), bradykinin (30 μM) or PDBu (1 μM) for 2 h. Stimulation of cells with either MCh or PDBu caused robust increases (approximately 4–5-fold) in levels of the c-Fos protein. In contrast, no increase in c-Fos was observed after bradykinin treatment of cells (Fig. 9D).

PDBu treatment causes a sustained phosphorylation of CREB (Fig. 6A) similar to that of MCh that we would expect to see translated into gene transcription. When assessing endogenous gene transcription using c-Fos a reporter this proves to be the case, however use of an endogenous pCRE-luciferase reporter construct fails to demonstrate PDBu-mediated gene transcription. The apparent contradictory results obtained for PDBu activation of endogenous versus exogenous genes may be due to the different time courses utilized in these assays. The 6 h incubation necessary for pCRE-Luc reporter gene transcription gives rise to the possibility that PDBu is down-regulating PKC at this time, thus explaining why no pCRE-Luc response is observed.

## 4 Discussion

In neuronal systems CREB has been shown to regulate a wide variety of key processes including development, synaptic plasticity, memory and neuronal survival decision-making [1,2,37,38]. In human SH-SY5Y neuroblastoma cells perhaps most effort has been made to understand the regulation and role of CREB in cell differentiation and neurite outgrowth. Thus, retinoic acid and brain-derived neurotrophic factor (BDNF) have been shown to increase CREB-dependent transcriptional activation and the pathways linking their cognate nuclear and tyrosine kinase receptors to CREB activation have been explored at least to some degree [39,40]. With respect to GPCRs which classically couple to G_i/o_ proteins, it has been shown that μ-opioid receptors can increase nuclear Ca^2+^/calmodulin leading to CREB phosphorylation [41], whereas nociceptin/orphanin FQ, an endogenous ligand for opioid receptor-like (NOP) receptor, causes an increase in CREB phosphorylation through a Ca^2+^/cAMP pathway in SH-SY5Y cells [42]. In the present study we demonstrate that the G_q/11_-coupled GPCRs, M_3_ mACh and B_2_ bradykinin, can stimulate CREB phosphorylation, and show that (similar to the increases in Ins(1,4,5)P_3_ and [Ca^2+^]_i_ stimulated by the M_3_ mACh and B_2_ bradykinin receptors [22,28]) the temporal profile of CREB phosphorylation is dependent on the initiating receptor. Thus, while the initial amplitude of the CREB phosphorylation response mediated by each receptor is similar, the B_2_ bradykinin receptor causes a transient Ins(1,4,5)P_3_/Ca^2+^/CREB response, whereas the M_3_ mACh receptor causes much more sustained peak-and-plateau type responses. Like other signalling modules involved in cell fate decision-making (e.g. ERK [43,44]) the duration as well as the amplitude of CREB phosphorylation may have implications for the transactivational properties of CREB. Thus, accruing data highlight the ability of neurones to decode information contained in amplitude, frequency, duration and locus differences in Ca^2+^ influx/mobilization [18–20,45], allowing CREB activity to be tightly regulated with respect to stimulus-specificity. Here we show that the sustained increase in phospho-CREB mediated by the M_3_ mACh receptor is capable of increasing CREB-dependent reporter gene transcription and c-Fos expression, whereas the transient signal generated by B_2_ bradykinin receptor activation, despite being of equivalent initial amplitude, is not sufficient to mediate these effects.

A number of protein kinases have the ability to phosphorylate CREB within its KID [3–12], therefore, we utilized a range of kinase-selective inhibitors, and other cell manipulations, to determine which are involved in M_3_ mACh and B_2_ bradykinin receptor signalling to CREB. By manipulating trans-plasmalemmal Ca^2+^ gradients in the SH-SY5Y cells, we were able to demonstrate that Ca^2+^ influx/mobilization is required for M_3_ mACh and B_2_ bradykinin receptor-mediated CREB phosphorylation. In addition, inhibition of the activity of the CaM-kinases resulted in approximately 50% inhibition of CREB phosphorylation mediated by either receptor. This suggests that one of the mechanisms by which Ca^2+^ signals lead to CREB phosphorylation is via the activation of the CaM-kinase family of proteins, which are known to phosphorylate CREB in neurones [4,46,47].

As well as the CaM-kinases, there are other potential effectors of Ca^2+^ signalling in the cells, including the Ca^2+^-dependent PKCα and βI isoenzymes that are expressed in SH-SY5Y cells [30]. Our data, using chronic phorbol ester exposure to down-regulate conventional and novel PKC isoenzymes, or pharmacological PKC inhibition, demonstrate a PKC requirement for normal CREB phosphorylation responses to both MCh and bradykinin. These data add to the growing body of evidence supporting a role for PKC as an important effector of CREB phosphorylation [48–50], however, it has yet to be elucidated whether PKC itself leads to the phosphorylation of CREB, or whether PKC in turn activates a downstream CREB kinase. To address whether the inhibitory effects of extracellular Ca^2+^ depletion on agonist-stimulated CREB phosphorylation are (partially) mediated via Ca^2+^-dependent PKC isoenzymes, the additivity of extracellular Ca^2+^ depletion and PKC inhibition were assessed. We observed a significantly greater inhibitory effect of combining these manipulations suggesting that Ca^2+^-independent (novel) as well as Ca^2+^-dependent (conventional) PKC isoenzymes may be involved in MCh- and bradykinin-mediated CREB activation.

CREB can also be phosphorylated by the ERK-regulated kinase, p90 ribosomal S6 kinase [7,8,51]. Here we have made an unexpected observation regarding the ERK/RSK-dependency of agonist-mediated CREB phosphorylation. Thus, while stimulation of the M_3_ mACh receptor causes a robust activation of ERK in SH-SY5Y cells, inhibition of this pathway (using the MEK1/2 inhibitor U0126) had no effect on the ability of MCh to cause CREB phosphorylation. However, in contrast, stimulation of the B_2_ bradykinin receptor caused only a modest and transient activation of ERK, but in this case U0126 significantly inhibited bradykinin-stimulated CREB phosphorylation (see Fig. 8). In addition, we have shown that while the inhibition of either ERK or PKC (through chronic phorbol ester treatment) activities each inhibit bradykinin-stimulated CREB phosphorylation by ≥50%, combined ERK/PKC inhibition had no greater effect than inhibiting either pathway alone. These data suggest that PKC and ERK may lie on a common upstream pathway linking B_2_ bradykinin receptor activation to CREB phosphorylation. PKC signalling to ERK has been reported in the literature [52,53]. Further work examining the profiles of PKC isoenzymic activation by MCh and bradykinin will be needed to establish why both M_3_ mACh and B_2_ bradykinin receptors exert PKC-dependent regulations of CREB, yet only one pathway proceeds via a partially ERK-dependent mechanism.

Previous work has provided strong evidence for a multiplicity of protein kinase input to CREB regulation with the relative contributions of the different kinases varying through the time-course of the CREB response [47,48,54]. Although in the present study we have implicated a number of kinases that act downstream of M_3_ mACh and B_2_ bradykinin receptors to regulate CREB, we have provided no evidence for their sequential recruitment to shape the CREB phosphorylation response. Indeed, our data suggest that CaM-kinase, PKC and/or ERK-dependent pathways are all important in initiating the response and other mechanisms (e.g. receptor desensitization) are likely to account for differences in the time-courses of CREB phosphorylation and transcriptional activation observed for the M_3_ mACh and B_2_ bradykinin receptors.

With respect to the connexion between CREB phosphorylation and activation, phosphorylation of CREB at Ser-133 is commonly assumed to be predictive of the activation of CRE-dependent transcription, however, several other post-transcriptional modifications of CREB may occur to influence the transcriptional activation state of CREB [2,55,56]. Using both an exogenous recombinant reporter construct and endogenous gene product we have demonstrated that M_3_ mACh receptor activation results in an increase in gene transcription. In contrast, the more transient increase in CREB phosphorylation caused by B_2_ bradykinin receptor activation did not translate into significant increases in either gene product. These data strongly suggest that sustained increases in CREB phosphorylation are essential for transcriptional activation in SH-SY5Y cells. It has been demonstrated previously that a sustained increase in intracellular Ca^2+^ is required for the initiation of gene transcription via CREB in both striatal neurons in response to metabotropic glutamate receptor activation [57] and cortical oligodendrocyte progenitor cells [48]. This might explain the differences in transcriptional ability for the two receptors studied here. Activation of the M_3_ mACh receptor results in the prolonged Ca^2+^ signal required to initiate transcription, whereas the B_2_ bradykinin receptor can only elicit a transient Ca^2+^ signal and associated CREB phosphorylation that may be insufficient to facilitate CRE-dependent transcriptional activity.

Finally, in neurones it is likely that metabotropic and ionotropic inputs will integrate to determine CREB phosphorylation and transcriptional activity [15–17,29,56]. While metabotropic and ionotropic inputs may activate CREB via parallel, independent pathways, it is more likely that modulatory cross-talk will occur. Thus, our group has demonstrated ionotropic effects on GPCR signalling in neurones [58] and provided new evidence that such interactions may be more complex than first thought by demonstrating that M_3_ mACh and B_2_ bradykinin receptor signalling can be differentially regulated by changes in membrane potential in SH-SY5Y cells [59]. However, here have shown that while K^+^-depolarization can cause a modest increase in CREB phosphorylation (Fig. 3), concurrent addition of MCh or bradykinin in the presence of increased extracellular K^+^ did not alter the CREB phosphorylation response to the GPCR agonist alone.

In conclusion, we have demonstrated that activation of both the M_3_ mACh and B_2_ bradykinin receptors lead to the phosphorylation of the transcription factor CREB via a primarily Ca^2+^-dependent mechanism in SH-SY5Y neuroblastoma cells. While both GPCRs appear to utilize PKC and Ca^2+^/CaM-kinase-dependent pathways, only the B_2_ bradykinin receptor additionally utilized an ERK1/2-dependent pathway. In contrast to the robust activation of both endogenous and exogenous indicators of transcriptional activation by the M_3_ mACh receptor agonist, the transient activation of Ca^2+^, PKC and ERK1/2 mediated by the B_2_ bradykinin receptor was an insufficient stimulus to bring about CRE-dependent gene transcription. These data demonstrate that while two different receptors may activate the transcription factor CREB via similar, overlapping pathways, translation of this signal into long-term phenotypic changes requires a particular kinetic activation profile. Furthermore, it will now be crucial to evaluate the interaction of neuronal-activity and G_q/11_-coupled GPCR-dependent CREB activation and ultimately gene expression in synaptically active primary neurones. The balance between protein kinase and protein phosphatase activity may depend not only on the intrinsic regulation of the GPCRs, but also the influence of synaptic activity on the localization of these enzymes and their possible scaffolding proteins.
